# Bridging the Gap Rather Than Filling the Entire Valley—Anatomic Insights When Treating the Medial Infraorbital Region

**DOI:** 10.1111/jocd.16582

**Published:** 2024-09-16

**Authors:** Francesco P. Bernardini, Brent Skippen, Raul Cetto, Mariana Calomeni, Sebastian Cotofana, Simone Ugo Urso, Ferdinando Paternostro, Morris E. Hartstein

**Affiliations:** ^1^ Oculoplastica Bernardini Genoa Milano Italy; ^2^ Private Practice Wagga Wagga New South Wales Australia; ^3^ Private Practice London UK; ^4^ Clinica Bravo Rio de Janeiro Brazil; ^5^ Department of Dermatology Erasmus Medical Centre Rotterdam The Netherlands; ^6^ Centre for Cutaneous Research Blizard Institute, Queen Mary University of London London UK; ^7^ Department of Plastic and Reconstructive Surgery Guangdong Second Provincial General Hospital Guangzhou Guangdong China; ^8^ Phlebology Center Bologna Italy; ^9^ Department of Experimental and Clinical Medicine, Anatomy and Histology Section University of Firenze Florence Italy; ^10^ Ophthalmic Plastic and Reconstructive Surgery Tel Aviv Israel

**Keywords:** cannula treatments, dermal filler, facial anatomy, filler, G‐Point Lift, intradermal injection, soft tissue, soft tissue fillers, SOOF, tear trough

## Abstract

**Background:**

The treatment of the medial infraorbital region also termed the tear trough has become increasingly popular by the use of soft tissue fillers in a minimally invasive approach using a cannula.

**Methods:**

A total of 246 tear troughs were injected and investigated originating from 123 study participants. The clinical outcome was evaluated 6 months after the treatment by independent observers based on standardized frontal images and the procedure was documented by ultrasound imaging.

**Results:**

On average, 0.26 (0.1) cc [range: 0.08–0.32] of soft tissue filler material was injected per tear trough. Tear trough depth was before the treatment rated as 2.12 (0.4), whereas after the treatment it was 1.15 (0.4) (*p* < 0.001). Hyperpigmentation score was 2.19 (0.4) before the treatment, whereas after the treatment it was 1.31 (0.5) (*p* < 0.001). Intraorbital fat pseudo‐prolapse severity was rated before the treatment 1.88 (0.7), whereas it was rated after the treatment 1.14 (0.3) (*p* < 0.001). Wrinkle severity of the lower eyelid was rated before the treatment 1.51 (0.6), whereas it was rated after the treatment 1.12 (0.3) (*p* < 0.001).

**Conclusion:**

The results of this retrospectively investigated case series revealed that the conducted injection technique for treating the tear trough for medial infraorbital hollowing with a cannula provided statistically significant clinical improvement with a limited adverse events profile. The technique utilized an injection approach which was perpendicularly oriented to the longitudinal axis of the tear trough thereby “bridging the gap instead of filling the entire valley.”

## 1 Introduction

The number of conducted minimally invasive soft tissue filler injections is constantly increasing due to its widespread acceptance and availability. The 2022 annual statistics, released by The Aesthetic Society, indicated that in the US between 2021 versus 2022, an increase of 13% occurred in the procedural count of soft tissue filler injections underscoring the popularity of this aesthetic intervention [1]. Hyaluronic acid‐based material is injected in specific facial regions resulting in a volumizing and a repositioning effect thereby ameliorating the sign of facial aging an improving facial volume and shape [2, 3, 4]. Despite that all facial regions can be targeted with soft tissue fillers, the most frequently targeted facial regions are lips, cheeks, and the infraorbital region. The latter can clinically be divided into the medially located tear trough and into the laterally located palpebromalar groove [5, 6, 7].

Anatomically, the tear trough is of special interest as this delicate facial region is the only one that contains only three fascial layers and therefore provides limited soft tissue coverage following the administration of filler material. These fascial layers are: skin, orbicularis oculi muscle (OOM), and periosteum. In addition, a recent study by Calomeni et al. [8] revealed that the angular vein travels inside the OOM and that the corresponding angular artery does not follow the path of the vein but is located more medially. This is in line with a previous publication by Gombolevskiy et al. [9], which revealed that in the tear trough the angular artery travels in the majority of the cases superficial to the OOM. These findings indicate that a safer injection location is the plane deep to the OOM and superficial to the maxillary periosteum of this region.

Various techniques have been suggested to treat the tear trough which include the use of a sharp‐tipped needle as well as the use of a blunt‐tipped cannula [10, 11]. A recent clinically focused literature review however provided no deeper insights which technique is better suited to achieve safer and satisfactory aesthetic results leaving room for new developments [12]. A detailed look into the underlying anatomy shows that the OOM is connected to the tear trough ligament and to the periosteum of maxilla of the tear trough [13, 14]. Due to the shape of the superficial nasolabial fat compartment the tear trough has an oblique and rectangular shape with its longitudinal axis pointing from medial superior to lateral inferior [15, 16]. This shape has prompted injection techniques to be directed from the anterior cheek (located lateral and inferior to the tear trough) cranially into the tear trough (located medial and superior) administering the product along the longitudinal axis of the tear trough [11]. Such an injection direction however (from the cheek into the tear trough) is parallel with the pathway angular vein and closely aligned with pathway of the angular artery. In addition, such injection techniques aim to volumize the tear trough along its longitudinal axis thereby potentially requiring more product to be used with is respective clinical consequences: swelling, surface irregularities, blue or yellow‐ish discolorations, and product migration.

It might be of potentially improved clinical benefit if the tear trough is targeted from a lateral approach crossing the tear trough axis perpendicularly as hereby less product is required to replenish the needed volume. Clinically, this would indicate that a dermal access point in the lateral infraorbital region is selected instead of a more caudally located point in the anterior cheek. The aim of this study is therefore to describe a case series of patients in which such an alternative injection strategy was performed targeting the tear trough from lateral but still applying the product into the supraperiosteal plane. The clinical outcome in this clinical case series was evaluated by independent observers and the procedure was documented by ultrasound imaging.

## 2 Methods

### 2.1 Study Design

The study was conducted between June 2021 and June 2022 and included consecutive patients of Oculoplastica Bernardini, Genoa, Italy. The procedures described in this study are part of the standard treatment protocol for medial infraorbital hollows and facial ultrasound scanning relevant for soft tissue filler injections.

No specific inclusion criteria were applied as this study enrolled consecutive patients following standard of care treatments with minimally invasive soft tissue fillers. Participants were not included into the study if they had undergone prior surgical facial interventions that could have influenced normal infraorbital anatomy or if they were unwilling to participate in the ultrasound scanning procedures.

The research adhered to Good Clinical Practice guidelines, complied with relevant Italian laws and regulations, and followed the ethical principles outlined in the Declaration of Helsinki. Ethical approval was not deemed necessary for this study, as it involved the retrospective analysis of standard facial aesthetic treatments for infraorbital hollows and ultrasound analyses, both routine procedures that are performed on a standard and regular basis at the study site.

### 2.2 Injection Procedure

**FIGURE 1:**
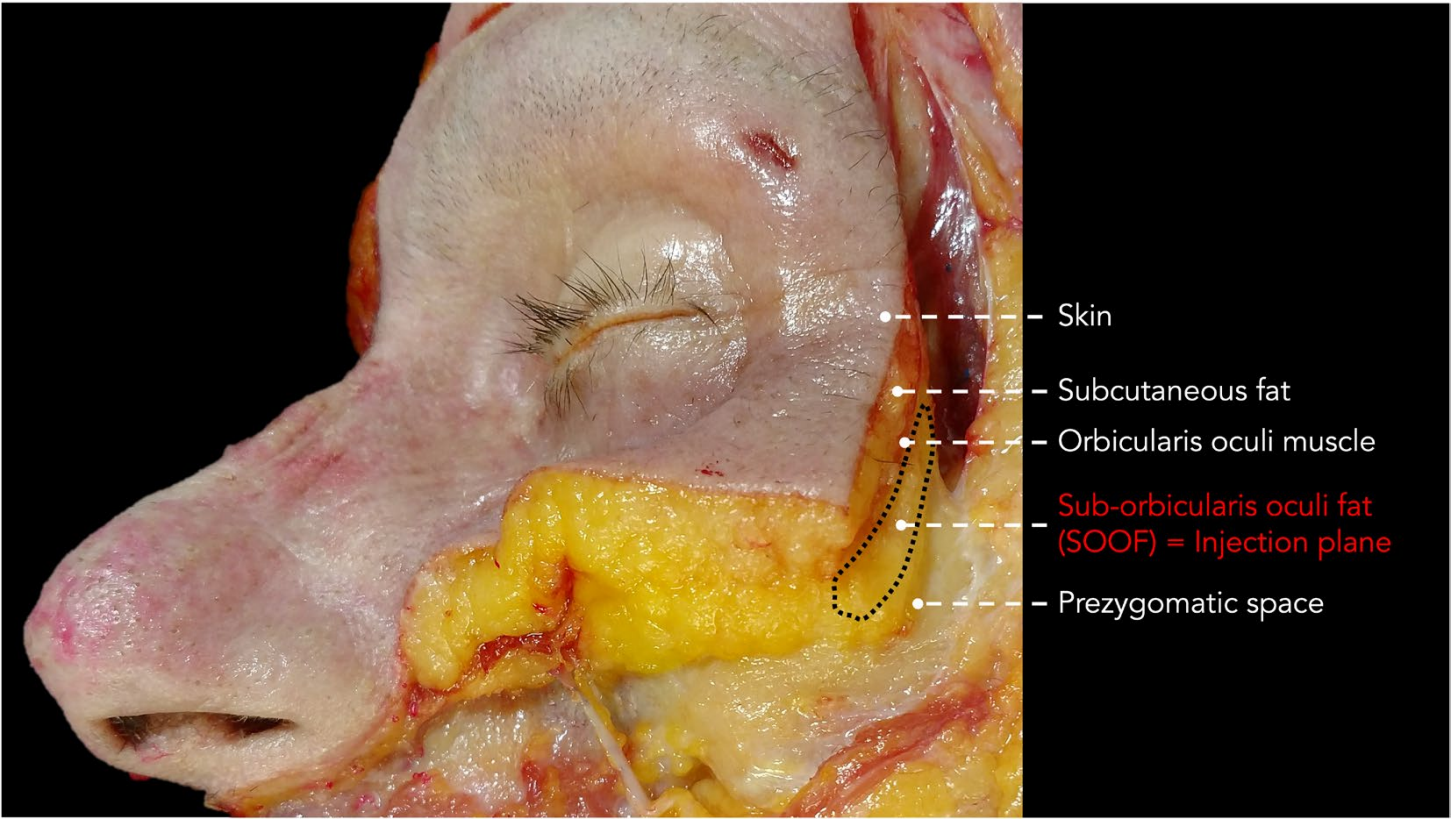
Dissection of cadaveric specimen showing the layers of the periorbital region. The sub‐orbicularis oculi fat (SOOF) compartment is targeted for the technique presented herein.

**FIGURE 2:**
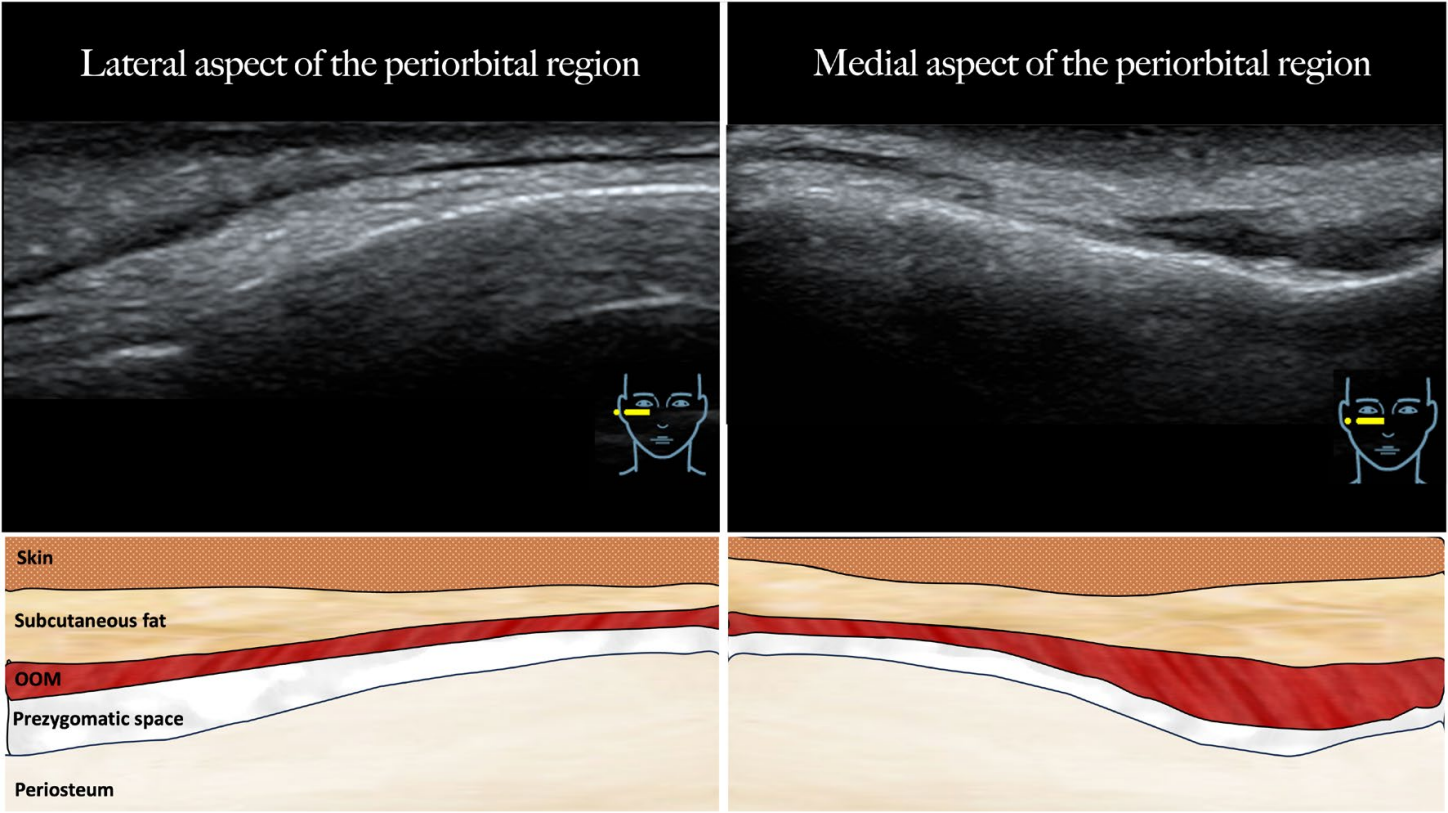
Ultrasound images the periorbital region with corresponding illustrations showing the layers in this region. The lateral aspect of the periorbital region is depicted on the left side, while the medial aspect is depicted on the right side of the image. The ultrasound transducer was placed in a horizontal fashion.

**FIGURE 3:**
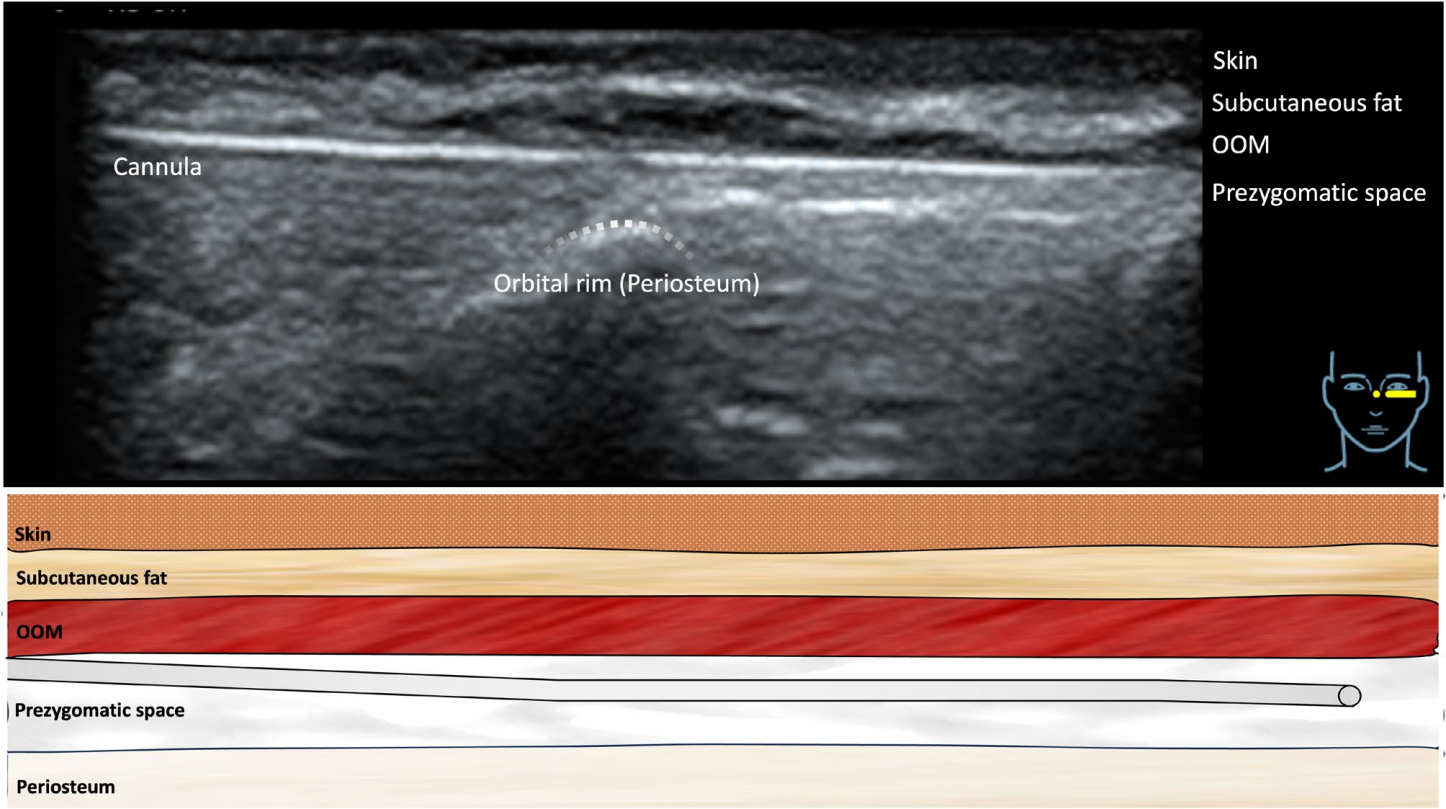
Ultrasound image showing the location of the cannula in the pregzygomatic space just superficial to the orbital rim (= periosteum). The ultrasound transducer was placed in a horizontal fashion.

**FIGURE 4:**
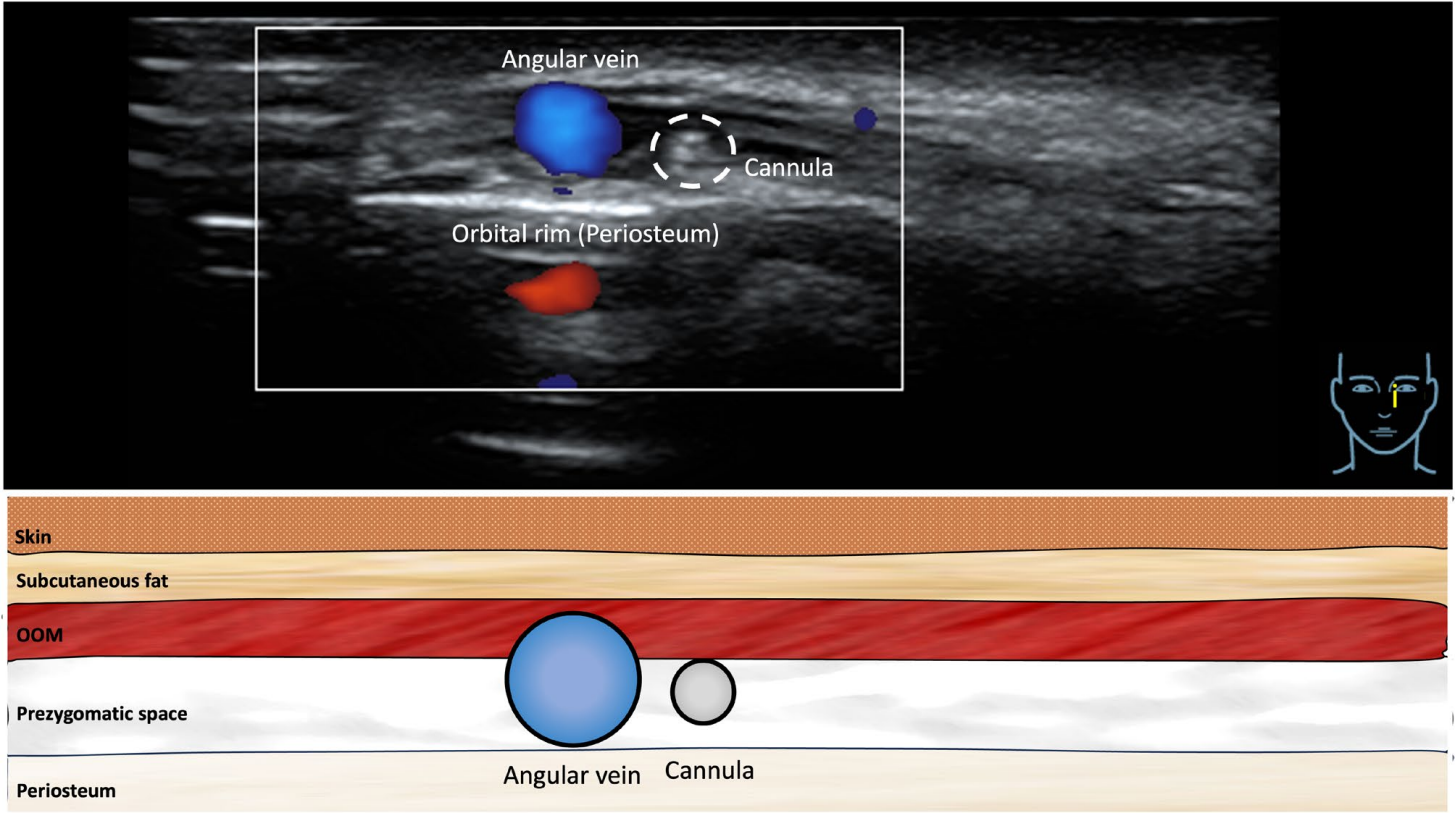
Ultrasound image showing the location of the cannula in the prezygomatic space right next to the angular vein and superficial to the orbital rim (= periosteum). The ultrasound transducer was placed in a vertical fashion.

The performed injection procedure utilized a 25G 50mm cannula (TSK) with a horizontal approach starting in the lateral infraorbital region. The dermal access point was previously described and relied on the skin surface projection of the SOOF located caudal to the ORL and cranial to the ZCL [17]. Following dermal puncture, the cannula was inserted deep to the OOM without establishing bone contact; this was achieved by immediate flattening of the cannula reaching the plane deep to OOM. This step was considered as the most crucial to not advance the cannula into the prezygomatic space but to remain inside the SOOF (Figures 1, 2, 3, 4).

**FIGURE 5:**
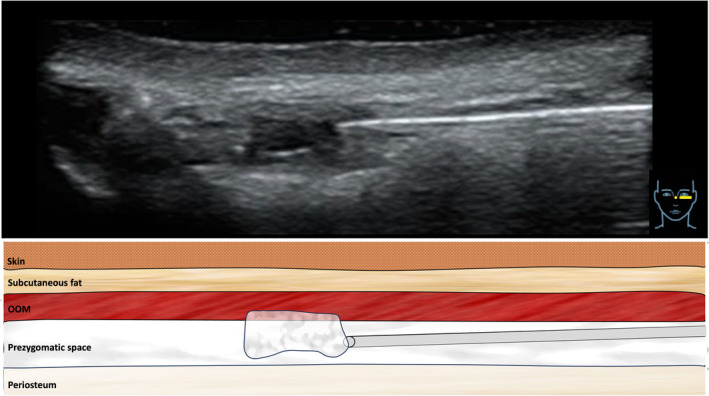
Ultrasound image showing the placement of soft tissue fillers in the prezygomatic space. The ultrasound transducer was placed in a horizontal fashion.

Once the desired plane was established without touching the bone but remaining deep to the OOM, the cannula was gently advanced medially. The depression forming the tear trough was crossed in a perpendicular angle to its longitudinal axis and upon retraction of the cannula soft tissue material was administered in a retrograde fashion. A total of 6–8 retrograde injection passes were conducted per facial side applying 0.02–0.04 cc per pass resulting in an average volume of 0.26 (0.1) cc [range: 0.08–0.32] of soft tissue filler material per facial side (Figure 5).

### 2.3 Ultrasound Imaging

Ultrasound scanning was conducted using advanced real‐time B‐mode grayscale imaging (average gain of 40) and color Doppler ultrasound devices (TE7 ultrasound machine from Mindray—China), equipped with high‐frequency transducers (ranging from 20 to 23 MHz). The ultrasound scanning procedures were performed by S.U.U., with at least 5 years of experience in facial soft tissue ultrasound and 30 years of experience in vascular ultrasound. Each participant was positioned at 45° upright angle. Periocular landmarks were meticulously marked, and a generous amount of ultrasound gel was applied to avoid skin deformation and soft tissue distortion. The ultrasound probe was suspended in the gel to prevent direct contact with the skin, thus ensuring accurate measurements. The probe was positioned in a horizontal scan along the inferior orbital rim and in a vertical scan along the medial canthal line. The depth of penetration was set to 1.5 cm for optimal visualization.

### 2.4 Clinical Assessment

Following a six‐month period after the initial treatment, standardized images of the clinical outcome were sent out to two independent expert raters (B.S. & R.C.) for image‐based evaluation. The following parameters were assessed in a pair‐wise reading process following a previously published rating system [18]:

- Depth of the tear trough: Measured as the distance in mm between the anterior lacrimal crest to the deepest location of the tear trough. Each millimeter is represented by one point, that is, 3 point on this scale represent a depth of 3 mm.
- Degree of hyperpigmentation (best to worst): 0 = no, 1 = mild, 2 = moderate, 3 = severe, 4 very severe.
- Degree of pseudo prolapse of intraorbital fat (best to worst): 0 = no, 1 = mild, 2 = moderate, 3 = severe, 4 very severe.
- Severity of lower eyelid rhytids (best to worst): 0 = no, 1 = mild, 2 = moderate, 3 = severe, 4 very severe.

Adverse events were monitored via patient diary or based on entries in the patients documents upon chart review.

### 2.5 Statistical Analysis

All calculations were performed using SPSS Statistics 27 (IBM, Armonk, NY, USA). Statistical significance was considered at a probability level of ≤ 0.05 to guide conclusions. Data are presented as mean and standard deviation (mean [SD]). Paired non‐parametric testing using Wilcoxon signed rank test were utilized to compare before and after scores.

## 3 Results

### 3.1 Clinical Sample Descriptions

A total of *n* = 246 tear troughs were injected and investigated originating from *n* = 123 study participants (6 males, 117 females) with a mean age of 36 years [range: 21–46].

### 3.2 Adverse Events

On average, 0.26 (0.1) cc [range: 0.08–0.32] of soft tissue filler material was injected per tear trough. The follow‐up interval for all patients included in this study was 6 months as per standard clinical practice. Patient safety was assessed by the recorded incidence and severity of adverse events (AEs) reported at all visits. Specific AEs evaluated from the diary included ecchymosis (bruising), edema (swelling), erythema (redness), and pain or tenderness. The most common AE was transient edema. All AEs resolved within two weeks. Bluish discoloration (the Tyndall Effect) was not observed in any case.

### 3.3 Clinical Outcome Scoring

Tear trough depth (as assessed by two independent observers; best to worst) before the treatment was rated as 2.12 (0.4) [range: 1–3], whereas after the treatment it was 1.15 (0.4) [range: 1–2], indicating a statistically significant improvement with *p* < 0.001.

Hyperpigmentation score (best to worst; 0–4) before the treatment was 2.19 (0.4) [range: 2–3], whereas after the treatment it was 1.31 (0.5) [range: 1–2], indicating a statistically significant improvement with *p* < 0.001.

Intraorbital fat pseudo‐prolapse severity (best to worst, 0–4) before the treatment was rated 1.88 (0.7) [range: 1–3], whereas after the treatment it was rated 1.14 (0.3) [range: 1–2], indicating a statistically significant improvement with *p* < 0.001.

**FIGURE 6:**
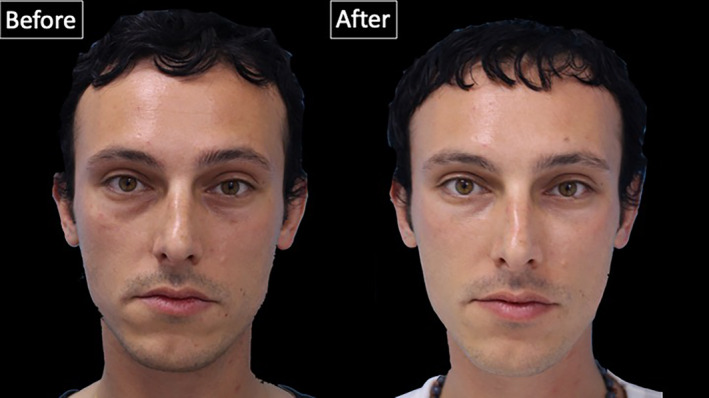
Photographs of a 35‐year old male patient before and after the injection technique proposed in this manuscript.

**FIGURE 7:**
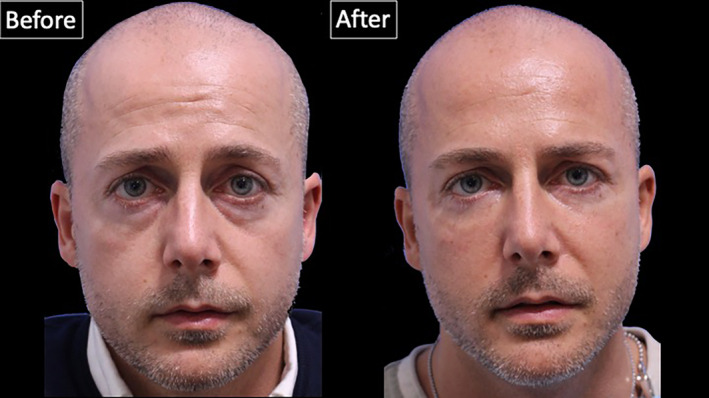
Photographs of a 38‐year old male patient before and after the injection technique proposed in this manuscript.

Wrinkle severity of the lower eyelid (best to worst, 0–4) before the treatment was rated 1.51 (0.6) [range: 1–3], whereas after the treatment it was rated 1.12 (0.3) [range: 1–2], indicating a statistically significant improvement with *p* < 0.001 (Figures 6 and 7).

### 3.4 Ultrasound Imaging

Post‐treatment ultrasound validation was conducted immediately after the treatment and at the six‐months follow examination. The administered product was identified in all investigated patients deep to the OOM and inferior to the tear trough ligament. No product was identified superficial to the OOM or within the lower eyelid soft tissues indicating the product was confined in the tear trough and did not migrate cranially or intraorbitally.

## 4 Discussion

The aim of this retrospectively investigated case series conducted in *n* = 123 consecutive patients was to evaluate the clinical outcome of a frequently performed cannula injection technique. The novel aspect of the performed injection technique is based on the geometrical evaluation of the tear trough which was identified to have an obliquely running axis from medial superior to lateral inferior. Whereas most frequently conducted injection techniques target the tear trough along its longitudinal axis, the presented cannula injection technique aims to treat the tear trough perpendicular to its longitudinal axis. This alternative approach is achieved by selecting the respective dermal access point in the lateral infraorbital region and by advancing the cannula horizontally in medial direction parallel to the orientation of the bony inferior orbital rim. Once the level of the medial vertical limbus line was reached during cannula advancement, a slight increase in resistance was perceived which corresponded to the penetration point through the zygomatico‐cutaneous ligament. It is of relevance to note that this location also corresponds anatomically to the pathway of the angular vein that travels however inside the OOM and is therefore located at a layer more superficial than the cannula advancement occurs. Once the tear trough was reached, 6–8 retrograde injection passes were conducted applying 0.02–0.04 cc per pass. This allowed for homogenous product distribution and a subtle filling of the tear trough until a smooth transition between the cheek and the lower eyelid was achieved. An average volume of 0.26 (0.1) cc [range: 0.08–0.32] of soft tissue filler material per facial side was required in this study resulting in a statistically significant (*p* < 0.001) improvement of the evaluated tear trough depth: from 2.12 (0.4) [range: 1–3] before the treatment to 1.15 (0.4) [range: 1–2] at 6‐months after the treatment.

The layer of product application was in the tear trough, the supraperiosteal plane located between the maxillary periosteum and the OOM. The ultrasound investigations conducted for the purposes of this case series revealed that the cannula was at the dermal access point inside the sub‐orbicularis oculi fat (SOOF) compartment and not in the deeper located prezygomatic space. Distinguishing between these two separate anatomic spaces is of importance for the performed cannula injection technique. The SOOF compartment allows for a smooth cannula advancement into the tear trough, whereas a blunt tipped cannula would experience more resistance inside the prezygomatic space upon movement. Additionally, the fascia separating the SOOF from the prezygomatic space (termed: midfacial extension of the superficial lamina of the deep temporal fascia) would significantly restrict the several conducted injection passes that helped to cross the tear trough and to evenly apply the product perpendicular to the longitudinal axis of the tear trough.

The conducted injection technique allowed for product placement caudal to the tear trough ligament, which corresponds to the separation between the palpebral and orbital part of the OOM and which also indicates the end of the cheek and the beginning of the lower eyelid. Positioning the product slightly caudal or in closest proximity to this ligament resulted in this study in a repositioning effect of the intraorbitally located nasal fat pad resembling the effects of a cantilever. This was evaluated at the 6‐months follow‐up visit as a statistically significant improvement (*p* < 0.001): from 1.88 (0.7) [range: 1–3] before the treatment to 1.14 (0.3) [range: 1–2] at 6‐months after the treatment.

Additionally, the volume deposition in the plane deep to the OOM resulted in a statistically significant improvement (*p* < 0.001) of the lower eyelid wrinkle status: from 1.51 (0.6) [range: 1–3] before the treatment to 1.12 (0.3) [range: 1–2] at 6‐months after the treatment. This provides clinical evidence for the sub‐OOM product application as the increased volume deep to OOM resulted in a stretching effect of the overlying OOM and as consequence in a reduced wrinkle status of the covering skin.

This study is not free of limitations, which should be noted here. The conducted injection technique is based on the experience of a single center and was conducted by one injector. It could be argued that in other and less experienced hands the clinical outcomes might be different and therefore more validation is needed. On the other side, it should also be mentioned that validity is increased in a case series like this if all treatments are performed by the same injector to assure consistency and reliability of the clinical outcomes evaluated at the 6‐months follow‐up examination. Another limitation of this study is the outcome assessment: the scoring system used in this study has limited validation and future studies will need to be conducted to identify their scientific merit.

Strengths of this study are however the large sample size consisting of *n* = 123 consecutive patients and the objective image‐based evaluation. The latter was conducted by other injection experts based on standardized two‐dimensional frontal images, which were not involved in the treatment process.

## 5 Conclusion

The results of this retrospectively investigated case series revealed that the conducted injection technique for treating the tear trough for medial infraorbital hollowing with a cannula provided statistically significant clinical improvement with a limited adverse events profile. The technique utilized an injection approach which was perpendicularly oriented to the longitudinal axis of the tear trough thereby “bridging the gap instead of filling the entire valley.” Future studies will need to expand on the results presented herein to validate the clinical outcomes and to expand the results to other patient collectives of different ethnic background and demographic profile.

## Author Contributions

F.P.B., B.S., R.C., M.C., S.C., S.U.U., F.P., and M.E.H. have made substantial contributions to conception and design, or acquisition of data, or analysis and interpretation of data. F.P.B., B.S., R.C., M.C., S.C., S.U.U., F.P., and M.E.H. have been involved in drafting the manuscript or revising it critically for important intellectual content and given final approval of the version to be published. Each author has participated sufficiently in the work to take public responsibility for appropriate portions of the content and agreed to be accountable for all aspects of the work in ensuring that questions related to the accuracy or integrity of any part of the work are appropriately investigated and resolved.

## Disclosure

S.C. is CEO of Cotofana Anatomy Corp., a company specialized in anatomical education.

## Conflicts of Interest

The authors declare no conflicts of interest.

## Supporting information


Video S1.

## Data Availability

The data that support the findings of this study are available from the corresponding author upon reasonable request.
