# Supplementary material for: Bridging the Gap Rather Than Filling the Entire Valley—Anatomic Insights When Treating the Medial Infraorbital Region
Source: J Cosmet Dermatol. 2024 Sep 16;24(1):e16582. doi: 10.1111/jocd.16582 (PMC11743227; doi:10.1111/jocd.16582)
Supplement: Supplementary file 1 — Video S1. [file JOCD-24-e16582-s001.zip › jocd16582-sup-0002-Caption.docx]

*Video legends*

*Video 1:* Demonstration of the injection technique proposed in this manuscript.
